# A Case Series on Whether Arthroscopic Bankart Repair Following Primary Traumatic Anterior Shoulder Dislocation or Recurrent Dislocation in Soldiers Has an Effect on the Postoperative Recurrence Rate

**DOI:** 10.7759/cureus.27655

**Published:** 2022-08-03

**Authors:** Tej P Gupta, Sanjay Rai, Amit Kale

**Affiliations:** 1 Department of Orthopaedics, Base Hospital, Guwahati, IND

**Keywords:** first-time shoulder dislocation, arthroscopy, arthroscopic repair, bankart repair, soldiers

## Abstract

Background

Anterior traumatic shoulder dislocation is very common among soldiers or any young population following injury, which is invariably treated by closed reduction. The dislocation when treated nonsurgically has a 71% high rate of recurrence. There is not much data available on the rate of recurrence when primary dislocation (first time) was treated by arthroscopic Bankart repair and in those who have recurrent (multiple) dislocations before surgery.

Aim

This study aims to report the postoperative recurrence rate in soldiers with first-time dislocation versus those with recurrent dislocations before surgery.

Study design

The present study is a level IV case series treatment study.

Methods

In this prospective study, 143 soldiers were included, of which 82 patients had first-time dislocation (F group) and 61 patients had recurrent dislocation before surgery (R group). The patients were evaluated and followed up for over three years. Nonabsorbable PEEK suture anchors (Chetan Meditech, India) were used for arthroscopic Bankart repair. The recurrence rates, Rowe scores, visual analog scale (VAS) scores, subjective shoulder values (SSVs), and satisfaction were compared. We also used the Simple Shoulder Test (SST) score for evaluation. The scores were recorded preoperatively and at three-year follow-up. The range of motion, postoperative function, recurrence rates, and return to pre-injury state was evaluated.

Results

A total of 143 patients were included, providing 97.3% follow-up at an average of 36 months. The postoperative recurrence rate was 19.7% in the F group and 58.3% in the R group (P < 0.001). The odds of postoperative recurrence were five times higher in the recurrent dislocation group (odds ratio (OR): 5.23).

Conclusion

Patients who underwent repair after first-time dislocation show a lower postoperative recurrence rate as compared with those who had multiple dislocations before surgery. It is prudent to advise early repair even after the first dislocation especially in young active soldiers to reduce the risk of postoperative recurrence.

## Introduction

Traumatic shoulder dislocation or instability is one of the most common sports-related injuries. These injuries affect the shoulder in various ways and often prevent the individual from returning to their pre-injury state. The glenoid labrum is the sheet anchor in maintaining the glenohumeral joint stability; as the bony glenoid fossa is shallow, a socket-deepening effect is created by the labrum for better stability, hence preventing dislocations.

Traumatic shoulder dislocation is very common in military soldiers due to their involvement in high-level contact sports, training, and other various kinds of military activities. While these soldiers need repair as early as possible to return to duty, there is no consensus, however, among surgeons on whether repairs conducted immediately after first-time dislocations achieve better outcomes and reduce chances of recurrence more effectively than repairs carried out after multiple episodes of dislocation. Waterman et al. [1] noted that younger patients possess an important risk factor for recurrence after Bankart repair; they further noted that patients who underwent arthroscopic repair had a significantly lower failure rate (4.5%) than those who underwent open repair (7.7%). Owens et al. [2] noted overall recurrence in 19 patients (31%); they also noted no significant benefits to open or arthroscopic repair. However, they observed that patients who had less than three episodes of subluxation before surgery showed better outcomes compared with those who underwent more episodes.

Many studies have shown the arthroscopic method of fixation to achieve favorable outcomes [3-5]. Moreover, arthroscopic surgery is preferred because of the disadvantages associated with open repair [6,7]. However, in a meta-analysis, Chen et al. [8] noted that, safety-wise, both arthroscopic repair and open Bankart repair were similar.

Although much advancement has occurred in the techniques of arthroscopic repair, there have been reports of failure rates as high as 30% [9]. As arthroscopic techniques have evolved over the last few decades, it is now prudent to reevaluate if these new advanced techniques have produced the expected results.

The aim of this present study is to report rates of recurrence in patients with primary dislocations (first time) versus recurrent dislocations (>2 dislocations before surgery) treated with arthroscopic Bankart repair.

## Materials and methods

In a prospective case study after institutional review board approval, a total of 143 patients were divided into two groups: the F group (82 patients with primary dislocations) and the R group (61 patients with recurrent dislocations). The study was conducted between February 2012 and December 2018.

The inclusion criterion was post-traumatic recurrent shoulder dislocation with a confirmed Bankart injury on MRI. The exclusion criteria were dislocations with glenoid or tuberosity fracture, large rotator cuff tear, capsular laxity, posterior instability, large Hill-Sachs lesion, and multidirectional instability. Furthermore, patients who had undergone an additional surgical procedure (capsular plication, remplissage surgery, or interval closure) during repair were also excluded.

Preoperative evaluation included history-taking, physical examination, and radiological evaluations. The number of dislocations, degree of instability, mode of injury, and time to return to the pre-injury level were recorded. Physical examination included range of motion measured and scaled with a goniometer, laxity assessment using the sulcus sign, load and shift, Jobe’s apprehension-relocation test, and anterior and posterior apprehension tests. Radiological evaluation included a plain X-ray of the shoulder (AP and axillary views) and an MRI. Subjective shoulder values (SSVs), visual analog scale (VAS) pain score, Shoulder Instability-Return to Sport after Injury (SIRSI) score, Rowe score, and satisfaction were used for comparison [10,11].

All surgeries were conducted by a team of two surgeons using a standardized technique with the patient under general anesthesia. Patients were operated on in a lateral position with the upper limb in 45 degrees of abduction and a 15-degree forward flexion with traction (3 kg weight). The shoulders to be operated on were evaluated for anteroposterior instability and inferior sliding. A standard single posterior and two anterior working portals were made. Initial diagnostic arthroscopy was performed to evaluate the status of the labrum, biceps tendon, rotator cuff, humeral head (Hill-Sachs lesion), and glenohumeral ligaments. Capsular laxity was examined thoroughly with a probe, and the drive-through sign was confirmed. After confirmation of anteroinferior instability due to Bankart lesion, capsulolabral tissues were freed with periosteum, mobilized, and shifted superiorly and laterally up to the glenoid rim using a periosteal elevator. Whenever possible, we used four 2.8-mm nonabsorbable PEEK suture anchors (Chetan Meditech, India); however, in some cases, only three anchors were used, depending on the size of the lesion. These were placed between the 1 o’clock and 5 o’clock positions for the right shoulder (7 o’clock and 11 o’clock for the left one). Postoperatively, all patients were placed in a shoulder immobilizer for four weeks.

Postoperatively, passive pendular exercises were started after the third day, active range of motion was started on the fourth week, and full return to activity and sports was allowed by the ninth month. Follow-ups at regular intervals (four weeks, eight weeks, three months, and every six months thereafter) were carried out for all patients in the outpatient department. We used the Simple Shoulder Test (SST) and University of California Los Angeles (UCLA) score for the evaluation of repair. The SST includes a series of 12 yes or no questions, with the maximum total score being 12 points; a higher score indicates better function. The UCLA score was used to measure the patient’s pain, function, strength, forward flexion, and patient satisfaction parameters. The maximum possible score is 35, and a higher score indicates better shoulder function. In the present study, a score of 34-35 points was considered excellent, 29-33 good, 21-28 mild, and 20 or lower poor.

**Figure 1:**
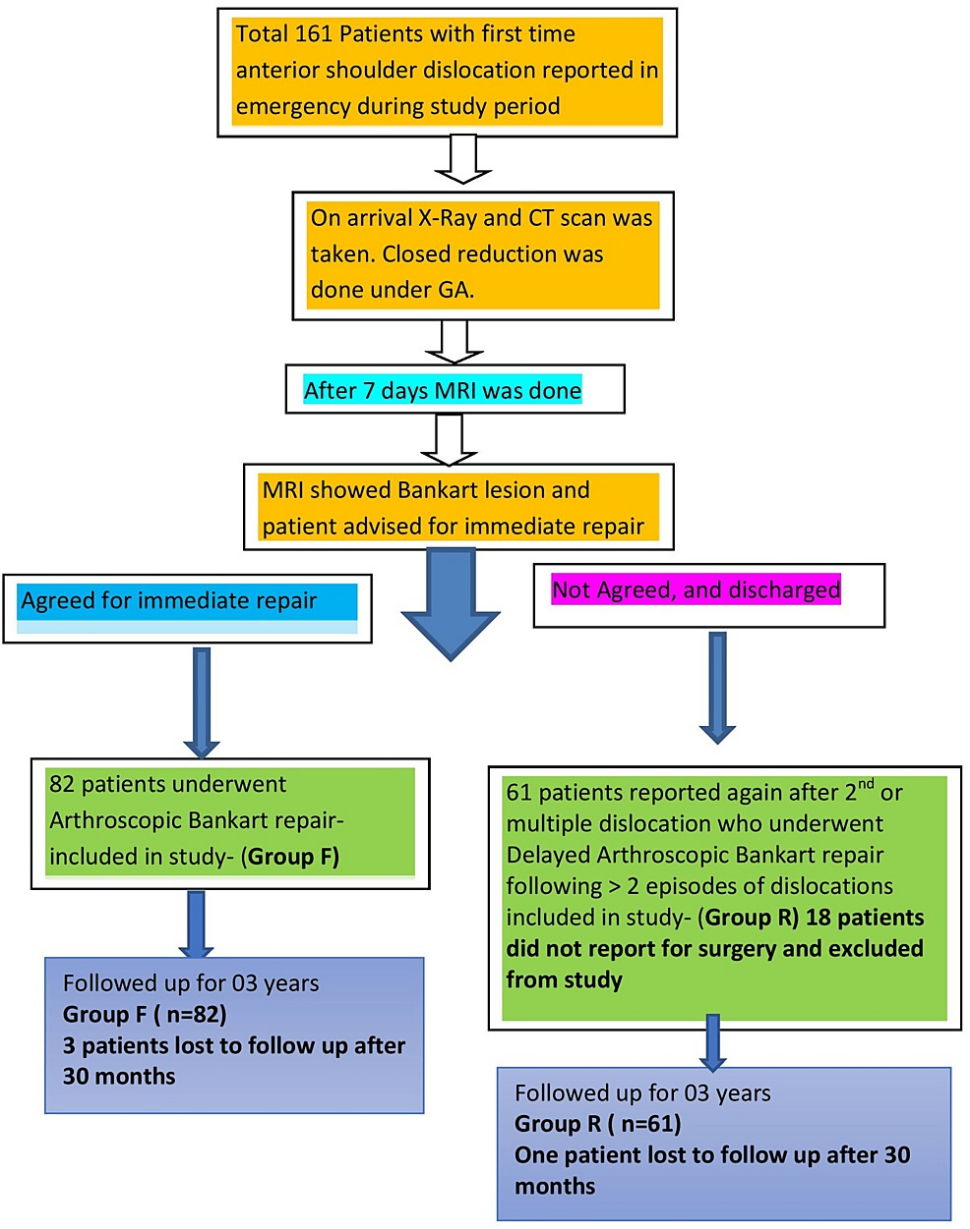
Study design

The study includes a total of 161 soldiers with emergency post-traumatic first-time anterior shoulder dislocation. However, 18 patients were excluded from the study as they were unwilling for repair after the detection of Bankart lesion on MRI conducted after closed reduction of acute dislocation (Figure 1).

Sample size calculation

For each group, to achieve a power of 80% and a level of significance of 5% (two-sided) and for detecting a true difference in means between the test and the reference group of 10 units, the total sample size comes to 72 (assuming equal size groups, 36 patients in each group), at 95% confidence interval (CI) (α = 0.05) with a population variance of 0.64° with 80% power (β = 0.2) (385). Considering that 10% of the patients failed to attend follow-ups during the study period, a sample size of 36 patients in each group was required (our sample included 82 patients in the F group and 61 patients in the R group).

Rehabilitation protocol

The rehabilitation protocol was the same for both groups. A shoulder brace was used on the operated shoulder for 21 days, and gentle passive movements without excessive abduction or rotation along with pendulum exercise were started postoperatively. Physiotherapy, such as abduction, flexion, and rotation, was gradually increased over the following 12 weeks. The soldiers were allowed to return to noncontact sports and restricted military training after 12 weeks; return to contact sports and full military training was allowed after six to nine months of surgery.

Statistical analysis

We used SPSS statistical software version 22 (IBM Corp., Armonk, NY, USA) for statistical analysis. Data were analyzed by comparing the preoperative versus postoperative UCLA scores utilizing the Wilcoxon matched pairs test, and data on preoperative and postoperative outcomes for the SST were compared using the unpaired t-test. P < 0.001 was considered significant. Further analysis was performed using paired Student’s t-test or Mann-Whitney test to compare means and Fischer’s exact test to test for categorical values because of the small sample size. Multivariate analytical tests were conducted to calculate the correlation between the various patient parameters and the outcome scores. Table 1 shows the demographical characteristics of the patients in the present study.

**Table 1:** Demographical characteristics of all patients who had arthroscopic Bankart repair

Characteristics	Number (%) or mean ± SD
Mean age (years)	35.12 ± 18.61
Sex	
Male	138
Female	5
Mean BMI (kg/m^2^)	26.4 ± 7.56
Number of patients included in the F group	82
Number of patients included in the R group	61
Smoking status	
Yes	13
No	130
Mean number of dislocations preoperatively (range) in the F group	1
Mean number of dislocations preoperatively (range) in the R group	6 (2-11)
Mean duration of operative time (range) in the F group	42.16 (23-115)
Mean duration of operative time (range) in the R group	61.42 (32-139)
Bony lesion visible on imaging	
Yes	35
No	108
Glenoid loss of contour	
Yes	29
No	114
Humeral head Hill-Sachs lesion	
Yes	9
No	134
Mean preoperative range of external rotation (range) in the F group	68.32 (48-90)
Mean preoperative range of external rotation (range) in the R group	74.54 (51-90)
Mean postoperative range of external rotation (range) in the F group	76.39 (57-90)
Mean postoperative range of external rotation (range) in the R group	83.12 (62-90)
Fully participated in the study	
Yes	139
No (lost to follow-up)	4 (lost after 30 months of follow-up)
Postoperative instability	
Yes	44
No	99
Underwent additional surgery	
Yes	24
No	119
Simple Shoulder Test score (N = 139, 4 lost after 16 months of follow-up)	12.7 ± 7.51

## Results

The demographical parameters of the soldiers are shown in Table 1, while the preoperative and intraoperative variables are presented in Table 2. After univariable analysis, we did not find any significant differences between the F and R groups on these variables. A univariable analysis comparison was conducted between soldiers with first-time (F group) and recurrent (R group) dislocations regarding their postoperative clinical outcomes, duration of follow-up, postoperative instability, requirement of additional surgery on the repaired shoulder, and Simple Shoulder Test score. It is worth noting that SST scores are available for 139 patients as four patients were lost to follow-ups after 30 months. We did not find any significant differences between the F and R groups on these variables.

Furthermore, we discovered that the recurrent dislocation group (R group) was at a higher risk for the development of postoperative instability compared with the first-time dislocation group (odds ratio (OR): 3.97, 95% CI: 1.83-8.56, P < 0.001). Similarly, the R group was also at an increased risk of requiring a second operation on the index shoulder (OR: 5.72, 95% CI: 2.12-16.38, P = 0.001). In addition, the R group had lower SST scores compared with the F group (13.62 versus 13.62, respectively) (P = 0.041) (Table 3).

**Table 2:** Comparative analysis of preoperative and intraoperative findings in patients with first-time versus recurrent dislocations Entries are presented as number (%) or mean ± SD. # Comparison based on data from 82 patients in the first-time dislocation group and 61 patients in the recurrent dislocation group. $ Wilcoxon rank-sum test with continuity correction. * Comparison based on data from 59 patients in the first-time dislocation group and 50 patients in the recurrent dislocation group. ** Pearson chi-square test with Yates continuity correction. & Comparison based on data from 82 patients in the first-time dislocation group and 61 patients in the recurrent dislocation group.

Parameters	F group (n = 82)	R group (n = 61)	P-value
Age at surgery (N = 143)^#^	35.12 ± 18.61	36.51 ± 18.32	0.672^$^
Body mass index (N = 143)^*^	21.42 ± 6.52	22.36 ± 8.13	0.682^$^
Sex (N = 143)			0.665^**^
Male (n = 138)	82	56	
Female (n = 5)	0	5	
Smoking status (N = 143)			0.833^**^
Yes (n = 13)	4	9	
No (n = 130)	78	52	
Bony lesion visible on imaging (N = 143)			0.712^**^
Yes (n = 35)	19	47	
No (n = 108)	63	14	
Humeral head Hill-Sachs lesion (N = 143)			0.636^**^
Yes (n = 9)	2	7	
No (n = 134)	80	54	
Glenoid loss of contour (N = 143)			0.671^**^
Yes (n = 29)	12	16	
No (n = 114)	68	45	
Number of anchors used in repair (N = 143)^&^	3.52 ± 1.42	3.49 ± 1.44	0.412^$^

**Table 3:** Comparative analysis of postoperative outcomes of patients with first-time versus recurrent dislocations * Recurrent dislocation odds compared with first-time dislocation odds. $ Wilcoxon rank-sum test with continuity correction. @ Pearson chi-square test with Yates continuity correction. & Comparison based on data from 139 patients (four patients lost to follow-up: three from the F group and one from the R group). CI: confidence interval

	Patients (number (%) or mean ± SD)		Odds ratio* (95% CI)	P-value
Parameters	F group (n = 82)	R group (n = 61)		
Postoperative instability			3.97 (1.83-8.56)	<0.001
Present	15	29		
Absent	67	32		
Additional surgery required			5.72 (2.12-16.38)	0.001^@^
Yes	6	18		
No	76	43		
Length of follow-up (months)	13.04 ± 7 14	14.09 ± 8.23	-	0.241^$^
Simple Shoulder Test score (n = 139)^&^	13.62 ± 62.19	11.18 ± 58.42	-	0.041^$^

A multivariable logistic regression analysis was carried out to show the effect of preoperative dislocation in both groups on postoperative instability and the requirement for a second surgery. Univariable analysis indicates a significant association between preoperative dislocation and postoperative instability and the requirement for a second surgery after eliminating for age, gender, the habit of smoking, and the number of suture anchors used in the Bankart repair. After controlling the abovementioned variables, the association between the preoperative dislocation group and the SST score became insignificant. We noted that the preoperative dislocation group was found to be the only variable with a significant association with postoperative instability. According to the multivariable model, the odds of postoperative instability were four times higher for patients who had more than two episodes of dislocation preoperatively compared with those with only one dislocation (OR: 4.39, 95% CI: 1.84-9.23, P = 0.0014). Additionally, the odds of requiring a second surgery were approximately five times higher in the recurrent dislocation group (R group) than in the first-time dislocation group (F group) (OR: 5.21, 95% CI: 2.19-23, P = 0.0012). These details are presented in Table 4 and Table 5. Small Hill-Sachs lesions or loss of bony glenoid did not have any significant impacts on the outcome.

**Table 4:** Multivariable logistic regression model for postoperative instability SE: standard error, CI: confidence interval

Characteristics	Coefficient	SE	Estimated odds ratio	95% CI	P-value
Intercept	0.123	1.23	1.14	0.097-13.3	0.896
Age (per one-year increase)	-0.078	0.0544	0.941	0.823-1.12	0.242
Male (versus female)	1.04	0.674	2.67	0.813-11.5	0.152
Smoker (versus nonsmoker)	0.78	0.83	2.31	0.437-12.4	0.411
Recurrent dislocation (versus first time)	1.47	0.402	4.39	1.84-9.23	0.0014
Anchors (per one-unit increase)	-0.089	0.15	0.091	0.647-1.36	0.613

**Table 5:** Multivariable logistic regression model for the requirement of a second surgery SE: standard error, CI: confidence interval

Characteristics	Coefficient	SE	Estimated odds ratio	95% CI	P-value
Intercept	-0.00184	1.92	0.988	0.0432-26.4	0.814
Age (per one-year increase)	-0.16	0.0843	0.896	0.721-1.09	0.215
Male (versus female)	1.04	0.674	2.67	0.813-11.5	0.152
Smoker (versus nonsmoker)	1.42	0.874	3.43	0.612-22.56	0.361
Recurrent dislocation (versus first time)	1.82	0.498	5.21	2.19-23	0.0012
Anchors (per one-unit increase)	-0.065	0.21	0.0987	0.687-1.27	0.742

At the final follow-up, we noted significant differences in the reported SIRSI scores between patients who underwent repair for primary instability and those who underwent it for recurrent instability (87.1 ± 28.4 versus 54.5 ± 19.3, P = 0.0025). We also recorded significant differences in the mean Rowe score, from 49.1 ± 7.8 preoperatively to 91.6 ± 5.12 at the final visit in the first-time dislocation group compared to 54.4 ± 8.3 preoperatively to 79.3 ± 16.4 at the final visit (P = 0.0241) in the recurrence instability group. However, we did not observe any significant differences in the VAS scores (2.5 ± 2.8 versus 1.9 ± 2.3, P = 0.0734) and SSVs (81.9 ± 16.4 versus 83.6 ± 20, P = 0.783). Moreover, satisfaction level was high in 72 (87%) patients in the F group and 48 (78%) patients in the R group (P = 0.0324). Patient-reported outcomes between both groups are shown in Table 6. We did not note any complications postoperatively in either group.

**Table 6:** Patient-reported outcomes between the F group and the R group

Parameters	F group (n = 82)	R group (n = 61)	P-value
VAS score	2.5 ± 2.8	1.9 ± 2.3	0.0734
Rowe score	91.6 ± 5.12	79.3 ± 16.4	0.0241*
SSV	81.9 ± 16.4	84.2 ± 19.7	0.783
SIRSI score	87.1 ± 28.4	54.5 ± 19.3	0.0025*
Satisfied	72 (87%)	48 (78%)	0.032*

We noted a total of 15 cases of instability postoperatively in the F group and 29 in the R group, which was significant (P = 0.0031). These findings are presented in Table 7.

**Table 7:** Recurrent instability

Parameters	F group (n = 15/82)	R group (n = 29/61)	P-value
Total recurrence	5	14	0.0031
Re-dislocation	3	3	0.642
Subluxation	1	3	0.825
Feeling of impending dislocation (apprehension)	6	9	0.635

## Discussion

Arthroscopic Bankart repair for recurrent glenohumeral instability has evolved considerably and is now globally the preferred method over open repair as it achieves better outcomes for the treatment of the Bankart lesion than the open technique [12]. An arthroscopic surgical repair has many advantages, such as being less invasive, preserving the subscapularis, and easing the management of concomitant pathologies. Being less invasive, it provides a more favorable range of motion and an early return to the pre-injury state.

After a review of the literature, we found that there is no general agreement on whether arthroscopic Bankart repair in first-time dislocations achieves better outcomes than repairs conducted in patients who have suffered multiple previous recurrent dislocation episodes preoperatively. In addition, reports on the rate of recurrence after repair also vary. In the present published data on the outcomes after arthroscopic repair, postoperative recurrence rates vary from 7% to 23% [13-15]. The results of the present study show higher postoperative recurrence rates than those previously reported, with 31% in the primary (first-time) dislocation group and 56% in the recurrent dislocation group. This increased rate of postoperative recurrence may be due to the younger age of the patients, as well as their involvement in contact sports and other military activities, which increase the chances of postoperative recurrence and the requirement for a second surgery [16].

In a recent meta-analysis, patients younger than 18 years with traumatic shoulder dislocation had a 71.3% recurrence rate when treated nonsurgically [17]; this is similar to the postoperative recurrence rate observed in our study (56%) among the recurrent dislocation group patients. This indicates that arthroscopic repair after recurrent dislocations presents similar results as those of the nonsurgically treated group. In the current study, we report postoperative recurrence as a re-dislocation/subluxation episode or an apprehension of dislocation.

We note that the need for repeat surgery in the recurrent dislocation group patients is 29% after arthroscopic repair. Additionally, Shymon et al. [18] found a recurrence rate of 27% in a retrospective cohort study of pediatric patients (average age of 17 years) treated using arthroscopic repair after anterior shoulder instability; the occurrence of recurrent instability is identical in our study among soldiers with first-time dislocations.

There is scanty data available on arthroscopic Bankart repair outcomes following one preoperative dislocation. Grumet et al. [19] conducted a systematic review to evaluate the recurrence rate in patients with first-time dislocation compared with those with recurrent instability; however, although 15 studies were included, no significant differences in recurrence between the groups were observed. In a study by Marshall et al. [20], 121 patients with 51 months of follow-up were divided into two groups: 53 patients in the recurrent dislocation group and 68 in the first-time dislocation group. They noted a postoperative 29% instability rate in the first-time dislocation group and a 62% instability rate in the recurrent dislocation group, which was considered significant (P < 0.001).

In a retrospective review of 50 patients who underwent isolated arthroscopic Bankart repair, Ono et al. [21] noted a 31.4% recurrence rate. Recently, some authors noted no significant difference in the recurrence rates following repair for first-time versus multiple dislocations before repair. In a study by Khalil et al. [22] on 20 young athletes with first-time dislocation and 20 with recurrent dislocation who underwent arthroscopic Bankart repair, no significant differences in postoperative recurrence rates were found among the patients. Similarly, Davey et al. [23] conducted a retrospective study on 100 athletes who underwent arthroscopic Bankart repair for primary instability pair matched with 100 patients who underwent the same procedure for recurrent instability and noted that there was no significant difference in the rate of recurrent instability after repair (10% versus 16%, respectively) (P = 0.29).

Our data indicate that soldiers with recurrent dislocations preoperatively, in comparison with soldiers with first-time dislocations, have a four times higher chance of postoperative instability and are five times more likely to need a second surgery. However, it is difficult to say if patients who underwent arthroscopic repair after a primary instability episode would not have experienced any further episodes had they been managed nonsurgically. As a result, it is not surprising that patients who underwent repair after their first dislocation had better outcomes than those who underwent repair following recurrent preoperative instability.

Olds et al. [24] noted that risk factors such as male gender, younger age (under 30 years), hyperlaxity, and contact sports increase the chances of recurrent instability. Our subset of the population (military soldiers) would have been at an increased risk for recurrent instability without repair because they are involved in high-impact sports and other military activities.

It is well known that bone loss in the glenoid and humeral head greatly influences shoulder instability [25-29]. In our study, based on radiographs, we did not observe any significant differences between first-time and recurrent group patients with regard to bone loss. Additionally, we also did not note a significant difference with respect to the occurrence of Hill-Sachs lesions, as we excluded the larger bone defect from our study.

Limitations of the present study

This study has some limitations as it was done in a specific subset of population, i.e., military soldiers, with no associated medical comorbidities. They were involved in various high-contact sports and training. We are not sure whether the outcome can be applied to the general population. Another limitation was its short-term follow-up period.

## Conclusions

The present study shows the benefits of arthroscopic repair after a primary (first-time) dislocation in a population of soldiers. Patients with primary (first-time) dislocations had lower odds of postoperative recurrence and re-surgery as compared with patients with recurrent dislocations preoperatively. In a soldier with shoulder instability, early arthroscopic repair is advised to lower the risk of postoperative recurrence and re-surgery.
